# 300. Clinical Experience with 16S Ribosomal RNA Gene PCR and Sequencing of Normally Sterile Body Fluids and Tissues in Pediatric Patients

**DOI:** 10.1093/ofid/ofae631.090

**Published:** 2025-01-29

**Authors:** Guyu Li, Christopher Reis, Rebecca M Kruc, Nicholas Streck, Elizabeth H Ristagno, Ziyuan Zhang, Matthew Wolf, James Gaensbauer, Robin Patel

**Affiliations:** Mayo Clinic, Rochester, Minnesota; Mayo Clinic, Rochester, Minnesota; Mayo Clinic, Rochester, Minnesota; Mayo Clinic, Rochester, Minnesota; Mayo Clinic, Rochester, Minnesota; Harvard T.H. Chan School of Public Health, Boston, Massachusetts; Mayo Clinic, Rochester, Minnesota; Mayo Clinic, Rochester, Minnesota; Mayo Clinic, Rochester, Minnesota

## Abstract

**Background:**

16S ribosomal RNA (rRNA) gene PCR and sequencing may serve as an adjunctive diagnostic tool in complex infections, identifying bacteria in patients on antibiotics whose cultures may be negative. Data on the optimal clinical settings for utilizing this type of testing are limited. This study reviews a single center's 16S rRNA PCR/sequencing experience in children to identify clinical syndromes and optimize specimen choice for higher yield, leading to testing of appropriate patients.

**Figure:**
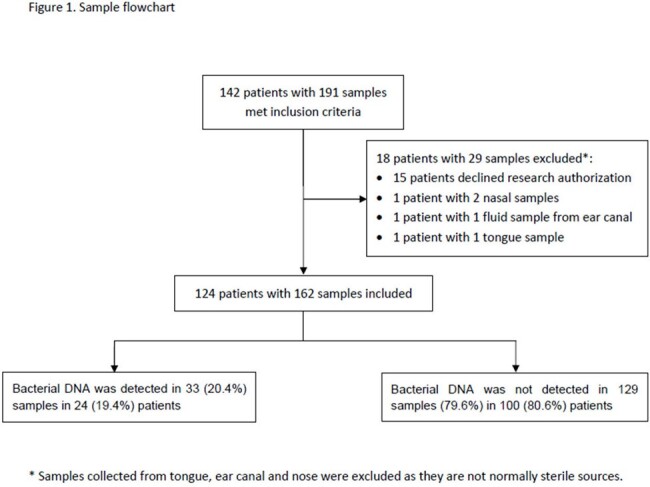

**Methods:**

A retrospective study was performed at Mayo Clinic, Rochester involving children aged 0 to 18 years whose normally sterile tissue or fluid samples underwent 16S rRNA gene PCR/sequencing. The assay involved an up-front real-time PCR assay, reported as negative or submitted to Sanger or next-generation sequencing depending on Ct values. Data was collected on patient characteristics and results of 16S rRNA gene PCR/sequencing and conventional tests. Impact on clinical decision-making was assessed as to whether the advanced molecular diagnostic changed antimicrobial therapy.

**Figure:**
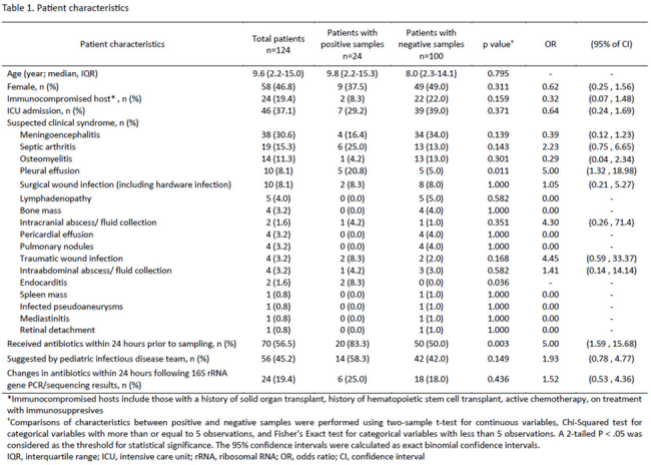

**Results:**

A total of 162 tests were performed on 124 patients, including 47% females (58/124) with a median age of 9.6 (range, 2.2-15.0) years. Overall, 33/162 (20%) of tests were positive in 24/124 (19%) patients. 58% of positive tests (19/33) were from samples with negative bacterial cultures. Cerebrospinal fluid was the most common specimen tested (n=38; 31%). Positive results were more likely from fluid compared to tissue samples (OR [95% CI]: 3.1 [1.3, 7.1], p=.007; OR [95% CI]: 0.3 [0.1, 0.8], p=.007, respectively). Testing was positive in 5/10 (50%) pleural fluids tested, a higher positivity rate than any other specimen type (OR [95% CI]: 5 [1.3, 19.0], p=.011). No significant changes in antibiotics were made within 24 hours of reporting positive 16S rRNA gene PCR/sequencing results.

**Figure:**
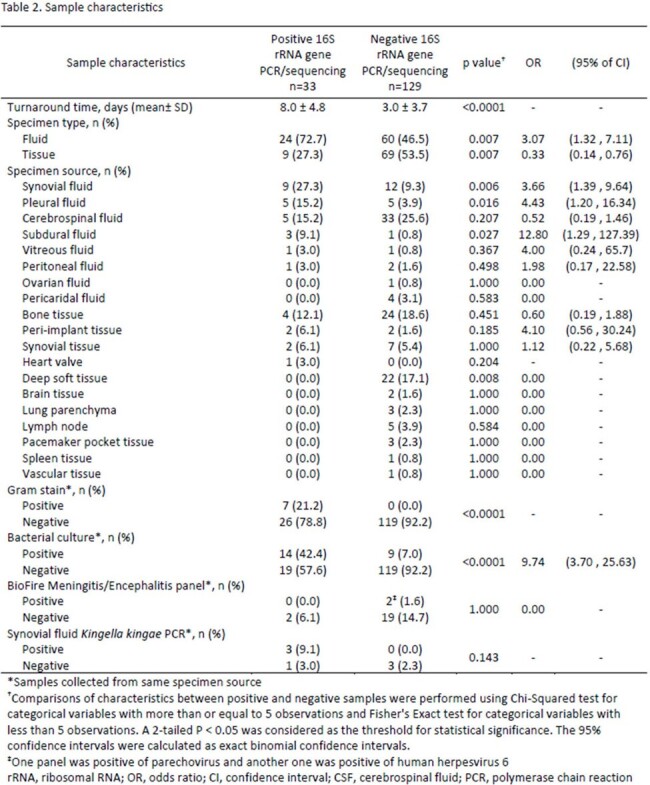

**Conclusion:**

This study highlights the importance of selecting appropriate sample types for 16S rRNA gene PCR/sequencing in pediatric patients. The assay is most likely to yield positive results in children with pleural effusion and in other fluid samples such as subdural and synovial fluid. Further implementation science research is needed to achieve impact on clinical practice.

**Figure:**
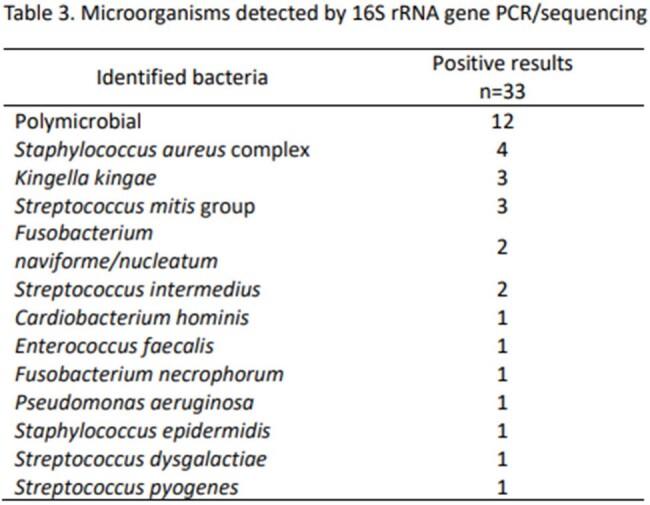

**Disclosures:**

**Robin Patel, MD**, a patent on Bordetella pertussis/parapertussis PCR issued, a patent on a device/method for sonication with royalties paid by Samsung to Mayo Clinic, a: See above|MicuRx Pharmaceuticals and BIOFIRE: Grant/Research Support|PhAST, Day Zero Diagnostics, Abbott Laboratories, Sysmex, DEEPULL DIAGNOSTICS, S.L., Netflix, Oxford Nanopore Technologies and CARB-X: Advisor/Consultant|Up-to-Date and the Infectious Diseases Board Review Course.: Honoraria

